# First Report of *Fusarium acuminatum* Causing Wilt Disease on *Vincetoxicum stauntonii* in China and the Control Strategy of Ethylicin via Membrane Damage and Oxidative Stress

**DOI:** 10.3390/jof12090642

**Published:** 2026-08-28

**Authors:** Hao Zhang, Qiaohuan Chen, Xiaoyu Yu, Honglu Wei, Cheng Chen, Dahui Liu, Mi Lei, Jinxin Li

**Affiliations:** 1 School of Pharmacy, Hubei University of Chinese Medicine, Wuhan 430065, China; zhanghao@stmail.hbucm.edu.cn (H.Z.); chenqiaohuan1@stmail.hbucm.edu.cn (Q.C.); 2023303010586@stmail.hbucm.edu.cn (X.Y.); 2023303010574@stmail.hbucm.edu.cn (H.W.); liudahui@hbucm.edu.cn (D.L.); 2 Hubei Shizhen Laboratory, Hubei University of Chinese Medicine, Wuhan 430065, China; 3 Qingqingmiao Ecological Agriculture Technology Development Co., Ltd., Wuhan 430065, China; chenchengqqm@126.com

**Keywords:** *Vincetoxicum stauntonii*, wilt disease, *Fusarium acuminatum*, biological characteristics, ethylicin, antifungal effects

## Abstract

*Vincetoxicum stauntonii* is a valuable traditional Chinese medicinal herb widely used in clinical practice for managing various respiratory disorders in China. However, a severe wilt disease recently prevailed in major *V. stauntonii* cultivation regions in Hubei Province from May to August 2025, resulting in an overall disease incidence exceeding 30%, with the maximum plant mortality rate exceeding 90% in severely affected fields. To clarify the causal agent of this emerging wilt disease and develop effective management strategies, symptomatic *V. stauntonii* plants were sampled from three representative commercial plantations. Pathogen isolation and purification were performed, followed by pathogenicity validation in accordance with Koch’s postulates. The assays confirmed that five isolates could induce typical wilt symptoms on healthy *V. stauntonii* plants. Based on morphological characterization and multilocus phylogenetic analysis using the concatenated internal transcribed spacer (ITS) region, translation elongation factor 1-alpha (*TEF1-α*), RNA polymerase II largest subunit (*RPB1*), and RNA polymerase II second largest subunit (*RPB2*) sequences, these five isolates were identified as *Fusarium acuminatum*. Biological characteristic assays showed that the causal agent grew optimally at 25 °C and pH 9.0, with soluble starch and peptone as the most suitable carbon and nitrogen sources, respectively. In vitro antifungal bioassays showed that ethylicin exhibited the strongest inhibitory effect against the pathogen, with a median effective concentration (EC_50_) value of 3.367 mg/L. Furthermore, greenhouse pot experiments validated that ethylicin exhibited significant protective and curative efficacy against *V. stauntonii* wilt disease. Mechanistic analyses further revealed that ethylicin disrupted plasma membrane integrity and induced excessive accumulation of intracellular reactive oxygen species (ROS) in *F. acuminatum*. To our knowledge, this study is the first formal report of *F. acuminatum*-caused wilt disease on *V. stauntonii*. Collectively, these findings provide a theoretical basis for the sustainable management of the emerging wilt disease of *V. stauntonii*.

## 1. Introduction

*Vincetoxicum stauntonii*, a perennial herb belonging to the Apocynaceae (formerly Asclepiadaceae), is a valuable traditional Chinese medicinal plant widely used in the clinical treatment of various respiratory disorders. Its dried rhizomes and roots serve as the primary medicinal components for alleviating bronchitis, cough, and asthma symptoms [1]. In recent years, the booming health industry and increasing demand for traditional Chinese medicines have driven the large-scale cultivation of *V. stauntonii*. Given the gradual depletion of wild germplasm resources, intensive artificial cultivation has become the primary means of ensuring a stable supply of medicinal materials. Hubei Province is the dominant national production region and the sole standardized cultivation base for *V. stauntonii* in China, accounting for more than 90% of the total commercial yield nationwide. Nevertheless, long-term monocropping and intensive cultivation have disrupted the field microecological balance, resulting in frequent disease occurrence. In particular, a severe wilt disease has emerged as a key constraint restricting the sustainable development of the *V. stauntonii* cultivation industry.

To date, only a limited number of fungal diseases have been documented in *V. stauntonii*, with *Fusarium tricinctum* and *Athelia rolfsii* causing seedling blight and southern blight, respectively [2,3]. Different from these recorded diseases, an unknown severe wilt disease occurred in *V. stauntonii* plantations from May to August 2025, causing considerable yield losses to local growers. Soil-borne fungal pathogens, including species of *Fusarium*, *Verticillium*, *Phialophora*, *Ophiostoma*, and *Plectosphaerella*, are the primary causal agents of crop wilt diseases [4,5,6]. Among these genera, *Fusarium* is widely recognized for its high species diversity, broad host range, and complex pathogenic mechanisms, capable of infecting more than 100 economically important crops [6,7]. Meanwhile, *Fusarium* species exhibit pronounced host specificity and geographical heterogeneity, which hinders accurate pathogen identification and targeted field management [6]. Furthermore, the long-term survival of wilt pathogens in soil and plant residues, combined with multiple transmission pathways mediated by soil, irrigation water, and farming operations, renders wilt diseases difficult to control worldwide [6]. Currently, systematic investigations into this emerging *V. stauntonii* wilt disease are still lacking. The definitive causal agent, biological characteristics of the pathogen, and targeted control strategies remain unclarified, representing an important research gap in the disease management of *V. stauntonii*.

In fungal molecular identification, the internal transcribed spacer (ITS) region of nuclear ribosomal DNA is widely used as a primary screening marker due to its broad applicability, extensive reference database, high success rate across diverse fungal taxa, and clear barcode gap between intra- and interspecific variation [8,9]. However, although ITS performs well for intergeneric discrimination, it exhibits limited resolution at the species-level within certain genera. The translation elongation factor 1-alpha (*TEF1-α*) is also regarded as one of the most informative loci for species-level identification within *Fusarium*, due to its high polymorphism and strong discriminatory power. Nevertheless, its highly variable introns can only be reliably aligned among members of a species complex or a few closely related taxa [10]. The largest (*RPB1*) and second largest (*RPB2*) subunits of RNA polymerase II are orthologous throughout the *Fusarium* genus, providing robust phylogenetic information at the species or near-species-level and allowing reliable sequence alignment across the entire genus [11]. These two markers possess robust resolution within *Fusarium*, their discriminatory power diminishes when applied to other fungal groups. Accordingly, multilocus phylogenetic analysis facilitates more accurate species-level identification of pathogens.

Current field management strategies for *V. stauntonii* wilt disease present notable practical bottlenecks. On the one hand, the unknown causal pathogen has limited the availability of targeted and precise control measures. On the other hand, local growers largely depend on experience-based, non-standard chemical fungicide applications. This practice results in inconsistent control efficacy, accelerated selection for fungicide resistance in the pathogen, and increased risks of pesticide residues [12,13,14]. Such non-standard pesticide use may compromise the quality and safety of *V. stauntonii* medicinal materials products and impede sustainable development of this industry. Under these conditions, there is a need to explore safe, effective, and eco-friendly alternative control approaches. Novel green fungicides, particularly botanical- and microbial-derived agents, have attracted increasing attention in contemporary plant disease management due to their high efficiency, low toxicity, ready biodegradability, and environmental compatibility [13,14]. These natural-product fungicides exhibit diverse and multi-site modes of action with a low risk of resistance, and show promise for disease management in medicinal and other cash crops. Accordingly, systematic evaluation of novel green fungicides and their preliminary inhibitory mechanisms is valuable for improving disease management and reducing chemical pesticide use [13,14].

This study aimed to clarify the causal agent of the emerging wilt disease affecting *V. stauntonii*. Diseased samples were collected from three commercial plantations for pathogen isolation and species identification. Tissue isolation, morphological characterization, multilocus molecular identification, and pathogenicity assays were integrated to verify the fungal pathogen responsible for this prevalent wilt disease in major *V. stauntonii* cultivation regions. Subsequently, the biological characteristics of the pathogen were systematically assessed by determining mycelial growth under varying temperatures, photoperiods, pH levels, and carbon and nitrogen source conditions. Furthermore, in vitro antifungal screening was performed on twelve fungicides covering chemical, botanical, and microbial agents. The most effective candidate with promising inhibitory activity was further evaluated for its in vivo protective and curative efficacy. To explore the preliminary inhibitory mechanisms, we analyzed the effects of the optimal fungicide on mycelial ultrastructure, plasma membrane integrity, intracellular reactive oxygen species (ROS) accumulation, and cellular electrolyte leakage. Collectively, these findings provide a theoretical basis for precise pathogen diagnosis, targeted field management, and rational application of eco-friendly fungicides, thereby facilitating the sustainable development of the *V. stauntonii* cultivation industry.

## 2. Materials and Methods

### 2.1. Sample Collection, Pathogen Isolation, and Purification

Diseased *V. stauntonii* plants showing typical wilt symptoms were collected on 21 May 2025, from three severely infected plantations in Xinzhou District, Wuhan, Hubei Province. All samples were placed in sterile kraft paper bags and immediately transferred to the laboratory for pathogen isolation. Pathogen isolation and purification were performed following a conventional tissue isolation protocol [15]. Briefly, sample surfaces were rinsed five times with sterile distilled water and air-dried. Tissue segments (0.5 cm × 0.5 cm) were excised from the margin of diseased and healthy stem tissues with a sterile scalpel. Tissue surfaces were disinfected with 75% ethanol (Sinopharm Chemical Reagent Co., Ltd., Shanghai, China) for 3 min [16], rinsed 5 times with sterile distilled water, and air-dried on sterile filter paper. The sterilized tissue blocks were placed on potato dextrose agar (PDA; Oxoid Ltd., Basingstoke, UK) plates supplemented with 0.1 mg/mL cephalosporin antibiotics and incubated at 25 °C in the dark. Fungal colonies emerging from the tissue explants were purified by single-hyphal-tip transfer on fresh PDA plates to obtain pure cultures. Purified isolates were maintained on PDA slants at 4 °C for short-term storage. For long-term preservation, 5 mm diameter mycelial plugs of pure cultures were transferred into 2 mL sterile centrifuge tubes containing 30% (*v*/*v*) glycerol (Sinopharm Chemical Reagent Co., Ltd., Shanghai, China) and stored at −80 °C.

### 2.2. Pathogenicity Assays

Pathogenicity of the isolates was verified via detached leaf inoculation and whole-plant inoculation assays, with six and nine biological replicates, respectively. For the detached leaf assay, two layers of sterile filter paper moistened with 5 mL of sterile distilled water were placed inside 90 mm Petri dishes [17]. Healthy *V. stauntonii* leaves were surface-rinsed with sterile water. Leaf centers were wounded with sterile needles to create 3 minor punctures, and a 5 mm diameter mycelial plug of the test isolate was placed on each wound. Leaves inoculated with sterile PDA plugs served as negative controls. All plates were sealed to maintain high humidity and incubated at 25 °C in continuous darkness, with disease development monitored daily.

For the whole-plant assay, uniform and healthy *V. stauntonii* plants were selected. The stem base of each plant was wounded 3 times with a sterile needle and then inoculated with a mycelial plug; control plants received sterile PDA plugs [2,3]. All inoculated plants were incubated in a growth chamber at 25 ± 2 °C with a 10 h light/14 h dark photoperiod. Longitudinal sections of the stem base were examined at 2, 3, 5, and 8 days post-inoculation (dpi), with symptom progression assessed daily.

### 2.3. Morphological Identification

The purified isolates were cultured on PDA plates for morphological characterization. Fresh hyphal tips from the colony margin were transferred onto 6 cm PDA plates and incubated at 25 °C in the dark. Colony morphology and pigmentation were documented after 8 days of incubation. Isolates were also subcultured on carnation leaf agar (CLA) and synthetic low-nutrient agar (SNA; Ruichu Biotechnology Co., Ltd., Shanghai, China) to induce sporulation for microscopic examination [15,16]. After 10 days, microscopic traits including hyphal morphology, septation, and sporulation traits were examined. Thirty conidia were randomly selected and measured using an IX73P1F inverted fluorescence microscope (Olympus, Tokyo, Japan) to determine conidial shape, length, and width.

### 2.4. DNA Extraction, PCR Amplification, and Sequencing

Total genomic DNA was extracted from 8-day-old fungal mycelia using a modified cetyltrimethylammonium bromide (CTAB; Phygene Biotechnology Co., Ltd., Fuzhou, China) method [18]. Four phylogenetic markers, including the ITS, *TEF1-α*, *RPB1*, and *RPB2*, were amplified by PCR. The corresponding primer pairs were ITS1/ITS4 [15], EF595F/EF1160R [19], Fa/G2R [16], and 5F2/7cR [20]. The primer sequences and PCR parameters are listed in Table 1. PCR products were resolved by 1% (*w*/*v*) agarose gel electrophoresis for 25 min and visualized under a gel imaging system to confirm target fragment amplification. Qualified PCR products were sent to Sangon Biotech (Shanghai, China) for Sanger sequencing; the resulting sequences were assembled and deposited in GenBank. The obtained sequences were compared against reference sequences in the NCBI nucleotide database using BLASTn tool (https://blast.ncbi.nlm.nih.gov/Blast.cgi, accessed on 6 September 2025). Furthermore, a phylogenetic tree was constructed based on concatenated ITS, *TEF1-α*, *RPB1* and *RPB2* sequences from five isolates, together with reference strains of *Fusarium* [21]. The concatenated phylogenetic tree was constructed using the maximum likelihood (ML) method in MEGA 12.0, with branch support assessed from 1000 bootstrap replicates.

### 2.5. Biological Characteristic Tests

#### 2.5.1. Evaluation of Temperature

Based on the consistent morphological, molecular, and pathogenicity results, BQ-3, which represented the highest proportion among all isolates during pathogen screening, was selected for all subsequent experiments. The mycelial growth rate was measured to determine the optimal growth temperature. Mycelial plugs (5 mm diameter) were punched from the margins of actively growing colonies and transferred onto fresh PDA medium. The cultures were incubated in the dark at 5 °C, 15 °C, 20 °C, 25 °C, 28 °C, 30 °C, 35 °C and 40 °C. Colony diameters were measured using the cross method [16], with three replicates per temperature treatment.

#### 2.5.2. Evaluation of pH

The optimal pH for mycelial growth was determined using the same experimental setup. Before autoclaving, the pH of the PDA medium was adjusted to 5, 6, 7, 8, 9, 10, 11 and 12 with 1.0 mol/L HCl or NaOH (Sinopharm Chemical Reagent Co., Ltd., Shanghai, China). Mycelial plugs (5 mm diameter) were inoculated onto pH-adjusted PDA plates and incubated at 25 °C in the dark. Colony diameters were recorded, with three replicates per pH treatment.

#### 2.5.3. Evaluation of Photoperiod

Freshly inoculated PDA plates were cultured at 25 °C under three photoperiod treatments: continuous light (24 h), alternating light/dark (12 h/12 h), and continuous darkness (0 h) [17]. Light intensity was maintained at 10,000 lux throughout the incubation period. Colony diameters were recorded, with three replicates per treatment.

#### 2.5.4. Utilization Preference of Carbon and Nitrogen Sources

To investigate the carbon and nitrogen source utilization preferences of the isolate, Czapek (CA) medium was used as the basal medium [22]. Sodium nitrate was used as the sole nitrogen source in the carbon utilization assays, and glucose as the sole carbon source in the nitrogen utilization assays [23]. Various carbon and nitrogen sources were individually added to evaluate the nutrient utilization of isolate BQ-3. Specifically, five carbon sources were individually added to the basal medium: fructose (30.00 g/L), maltose (28.50 g/L), soluble starch (27.00 g/L), sucrose (28.50 g/L), and glucose (30.00 g/L). Similarly, five nitrogen sources were also individually added: ammonium sulfate (2.33 g/L), ammonium chloride (1.89 g/L), peptone (3.41 g/L), sodium nitrate (3.00 g/L), and glycine (2.65 g/L) [23]. Mycelial plugs (5 mm diameter) were placed at the center of plates containing the respective carbon or nitrogen sources, and incubated at 25 °C in the dark. Final colony diameters were measured, with three replicates per treatment.

### 2.6. Sensitivity Screening of Fungicides

#### 2.6.1. Initial Screening of Various Fungicide Types

The antifungal activity of selected fungicides against strain BQ-3 was evaluated. Five chemical, four botanical, and three microbial-based fungicides were included. All fungicides except for the *Trichoderma harzianum* and *Bacillus subtilis* formulations were evaluated using the mycelial growth rate method [16]. Fungicides are listed in Table S1; each fungicide was added into sterilized PDA at the manufacturer-recommended field concentrations. Mycelial plugs (5 mm in diameter) excised from the colony margin were placed at the center of fungicide-amended PDA plates. PDA plates without fungicide served as blank controls. Colony diameters were measured using the cross method, and the mycelial growth inhibition rate was calculated accordingly. Each treatment was performed in three biological replicates.

The antifungal activity of microbial fungicides was evaluated using the plate confrontation method [24]. Sterile circular filter paper discs (5 mm in diameter) were soaked in suspensions of *T. harzianum* (6 × 10^5^ CFU/g) and *B. subtilis* (2 × 10^7^ CFU/g). Four filter paper discs were placed equidistantly in a cross pattern, 20 mm from the plate center. A 5 mm mycelial plug of the pathogen was then placed at the center of each PDA plate. Filter paper discs soaked in sterile water served as blank controls. All plates were incubated at 25 °C in the dark. Colony diameters were measured using the cross method, and the mycelial growth inhibition rate was calculated according to the formula [25]: i (%) = [(a_control_ − a_treatment_)/a_control_] × 100, where i represents the mycelial growth inhibition rate, a_control_ and a_treatment_ denote the hyphal area of the control and treatment groups, respectively. Each treatment had three biological replicates.

#### 2.6.2. Determination of EC_50_ Values of Four Candidate Fungicides

Based on the initial screening, four fungicides with stronger antifungal activity, namely carbendazim, carvacrol, osthole, and ethylicin were selected for concentration gradient assays. Five concentrations were tested for each fungicide (Table S2). Inhibition rate and median effective concentration (EC_50_) were calculated to quantify the antifungal efficacy [26]. Linear regression analysis of log concentration versus inhibition rate was performed using IBM SPSS Statistics 27.0. The regression equation, correlation coefficient (R^2^), 95% confidence intervals, and EC_50_ values were calculated. The inhibition rate was calculated as follows [27]: inhibition rate (%) = [(d_control_ − d_treatment_)/(d_control_ − 5 mm)] × 100, where d_control_ and d_treatment_ denote the average colony diameters of the control and treatment groups, respectively.

#### 2.6.3. The Efficacy of Ethylicin in Controlling *V. stauntonii* Wilt Disease

Given the significant inhibitory activity of ethylicin against the pathogen responsible for *V. stauntonii* wilt disease, pot experiments were conducted to evaluate its in vivo control efficacy. To evaluate the protective and curative effects of ethylicin, three groups were designed with consistent timing for pathogen inoculation, including the blank control group, prevention group, and treatment group [28]. Briefly, the stem base of each plant was wounded three times with a sterile needle, and a 5 mm mycelial plug was placed on the wound sites for inoculation [26]. In the prevention group, 5 mL of ethylicin solution (3.367 mg/L, EC_50_) was sprayed onto the wounds 24 h prior to inoculation with *Fusarium acuminatum* strain BQ-3 [28]. The treatment group received the same solution 24 h after inoculation. The control group received an equal volume of sterile water. All plants were maintained in a controlled growth chamber at 25 ± 2 °C with a 10 h light/14 h dark photoperiod. Each experimental group comprised ten individual plants.

Disease severity was assessed 8 days after treatment using a modified 0–5 disease index scale as described in Sultana et al. and Macioszek et al. [29,30]. Grade 0: healthy plants without symptoms. Grade 1: minor lesions at the stem base. Grade 2: mild wilting of apical buds with lesion expansion. Grade 3: curled and wrinkled leaves with pronounced stem-base lesions. Grade 4: withered and yellowed leaves with lesions encircling the entire stem base. Grade 5: plant mortality. The disease index was calculated as follows [31]: disease index (%) = [∑(n_i_ × v_i_)/(n × v_max_)] × 100, where n_i_ denotes the number of plants at each disease grade, v_i_ denotes the corresponding disease grade value, n denotes the total number of plants investigated, and v_max_ denotes the highest disease grade.

### 2.7. Analysis of the Preliminary Antifungal Mechanism of Ethylicin

#### 2.7.1. Scanning Electron Microscopy (SEM) Observation

Fungal colonies grown on PDA medium containing ethylicin at 0.5 × EC_50_, EC_50_, and 2 × EC_50_ for 8 days were cut into small pieces (5 mm × 5 mm). The samples were fixed with 2.5% (*v*/*v*) glutaraldehyde at 4 °C for 24 h. After fixation, the samples were rinsed three times with pre-cooled sterile phosphate-buffered saline (PBS; Solarbio Life Sciences, Beijing, China), with each rinse lasting 10 min. The samples were then dehydrated in a graded ethanol series (30%, 50%, 70%, 80% and 90%), 15 min per step. Final dehydration was performed in absolute ethanol for 45 min [27,31]. The dehydrated specimens were freeze-dried in a vacuum freeze dryer. All specimens were sputter-coated with gold. Finally, mycelial morphology was examined using an Apreo 2S LoVac SEM (Thermo Fisher Scientific, Waltham, MA, USA) at 5 kV.

#### 2.7.2. Determination of Cell Membrane Integrity and Intracellular Reactive Oxygen Species (ROS) Level

Four mycelial blocks (5 mm in diameter) were inoculated into 200 mL potato dextrose broth (PDB; Solarbio Life Sciences, Beijing, China) and incubated at 25 °C in the dark with shaking at 150 rpm for 4 days. The mycelia were then treated with ethylicin at 0.5 × EC_50_, EC_50_, and 2 × EC_50_; PDB without fungicide was set as the blank control [31]. After 12 h, mycelia were collected, rinsed three times with pre-cooled PBS (pH 7.2–7.4), and resuspended in fresh PBS [25]. Subsequently, membrane integrity was evaluated by propidium iodide (PI; Biosharp Life Sciences, Shanghai, China) and fluorescein diacetate (FDA; Macklin Biochemical, Shanghai, China) double staining [32,33]. Samples were simultaneously stained with PI-FDA in 1 mL PBS containing 5 μL of PI (1 mg/mL) and 2 μL of FDA (5 mg/mL) for 20 min at room temperature in the dark [31]. After staining, the samples were washed three times with pre-cooled PBS in the dark. PI and FDA signals were detected using an inverted fluorescence microscope at excitation/emission wavelengths of 535/617 nm and 490/520 nm, respectively. Intracellular ROS levels were detected using the 2′,7′-dichlorodihydrofluorescein diacetate (DCFH-DA; Solarbio Life Sciences, Beijing, China) staining [34,35]. Samples were stained with 10 μmol/L DCFH-DA at room temperature in the dark for 30 min. After staining, the samples were washed three times with pre-cooled PBS in the dark. The samples were then detected and photographed under the same microscope at excitation /emission wavelengths of 488/525 nm.

#### 2.7.3. Measurement of Cell Membrane Permeability and Leakage of Intracellular Nucleic Acids and Proteins

Mycelia were collected as described in Section 2.7.2. Fresh mycelia (1.0 g) were transferred to a 50 mL sterile centrifuge tube containing 30 mL of sterile water. Cell membrane permeability was assessed by measuring the electrical conductivity of mycelial suspensions at 0, 2, 4, 6, 8, and 10 h, with slight modifications [31,32]. After 10 h, the mycelial suspensions were centrifuged at 12,000 rpm for 10 min and filtered through a 0.22 μm filter. The absorbance of the supernatants was measured at 260 nm and 280 nm to quantify nucleic acid and protein leakage, respectively. All experiments were performed in triplicate. Relative electrical conductivity was calculated as: relative electrical conductivity (%) = [(c_t_ − c_0_)/(c_max_ − c_0_)] × 100, where c_t_ represents the electrical conductivity measured at each time point, c_0_ is the initial electrical conductivity at 0 h, and c_max_ is the maximum electrical conductivity after boiling for 10 min and cooling to room temperature.

### 2.8. Data Statistics and Analysis

All data were expressed as the mean ± standard deviation (SD). Statistical analyses and graph plotting were performed using GraphPad Prism 10.1.2 software. One-way analysis of variance (ANOVA) followed by Tukey’s multiple comparison test was adopted to compare the mean values among different treatments. Significant differences between means were indicated by different lowercase letters (*p* < 0.05). All experiments were conducted in at least three independent biological replicates.

## 3. Results

### 3.1. Field Disease Investigation and Pathogenicity of Isolates

**Figure 1:**
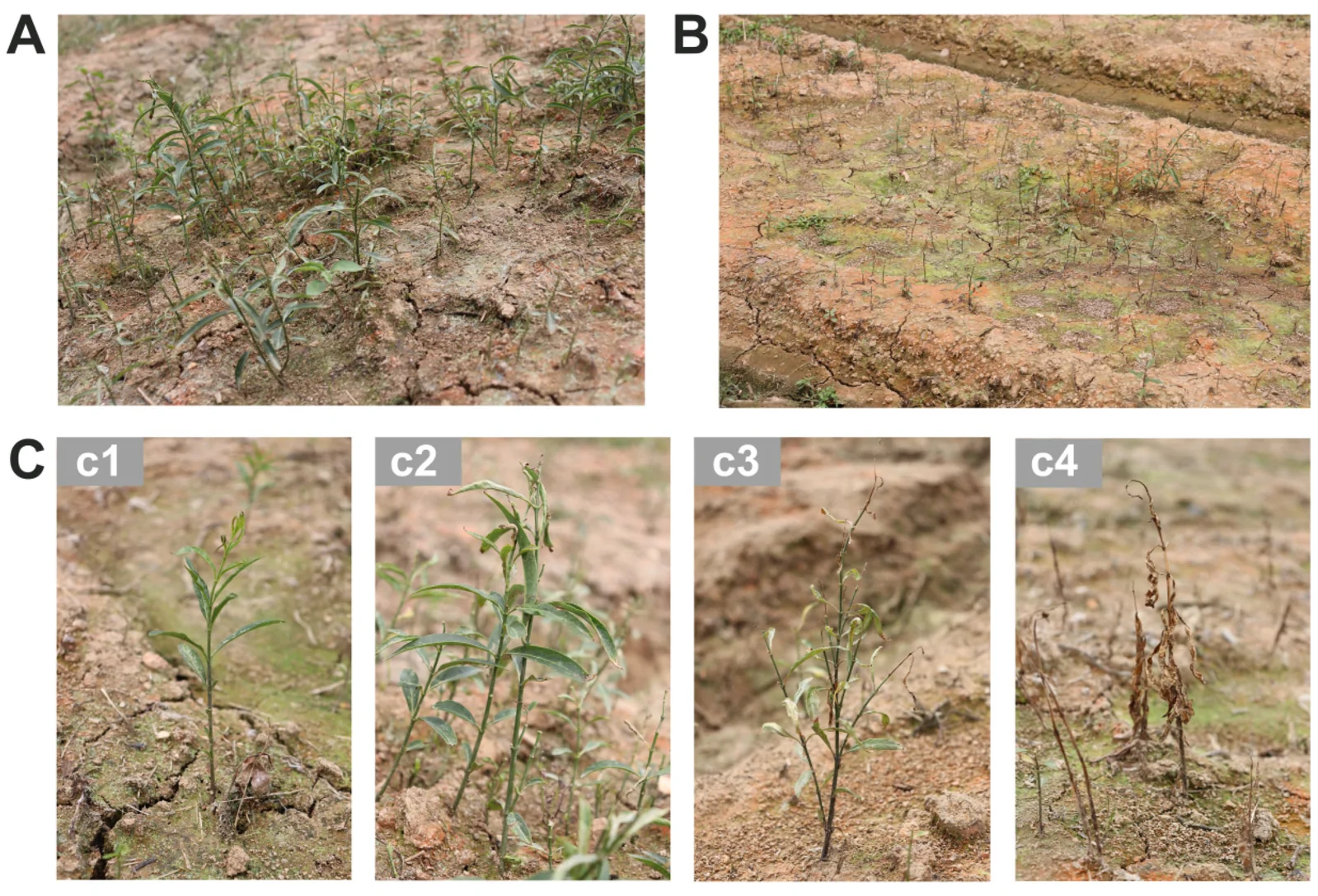
The field symptoms of wilt disease on *Vincetoxicum stauntonii*. (**A**) Early field symptoms. (**B**) Late field symptoms. (**C**) The progressive symptom development of *V. stauntonii* wilt disease.

Field surveys conducted in the core commercial cultivation regions of *V. stauntonii* in Wuhan, Hubei Province, revealed a severe outbreak and epidemic of wilt disease from May to August 2025. Meteorological data for this period (Figure S1) showed that both the mean temperature and total precipitation reached their annual maxima. Therefore, we speculate that high temperature and high humidity may be risk factors for disease epidemics. Three fields were surveyed using a five-point sampling method, with 30 plants examined in each field. Disease incidence was assessed by curled leaves and apical wilting, and mortality was determined by extensive defoliation, stem wilting, and desiccation. Overall field disease incidence exceeded 30%, and plant mortality rate reached over 90% in heavily infected plots (Figure 1A,B). Continuous symptom observation revealed a typical progressive pattern of *V. stauntonii* wilt disease. Early-stage symptoms mainly included wilting in apical young leaves and branches, which gradually progressed downward. At the middle stage, most leaves showed shriveling, wrinkling, and curling. In the late stage, systemic wilting of above-ground tissues occurred, followed by progressive leaf abscission as well as severe desiccation, dry rot, and necrosis of stems and leaves (Figure 1C).

To identify the causal agent of *V. stauntonii* wilt disease, 187 isolates were obtained from 15 severely diseased plants. More than 80% of the isolates produced red pigments, suggesting their dominance in diseased tissues and potential association with the wilt disease. Five representative pigment-producing isolates, designated BQ-1 to BQ-5 were selected for pathogenicity tests. In the detached leaf assay, mycelial plugs germinated and produced visible hyphae at 1 dpi. At 3 dpi, rapid hyphal proliferation and tissue invasion were observed, forming distinct grayish-black lesions at the inoculation sites. Lesions expanded outward at 5 dpi and developed into large necrotic areas by 8 dpi. Typical symptoms were yellowish-brown necrotic lesions with water-soaked black centers, whereas control leaves remained asymptomatic (Figure S2).

**Figure 2:**
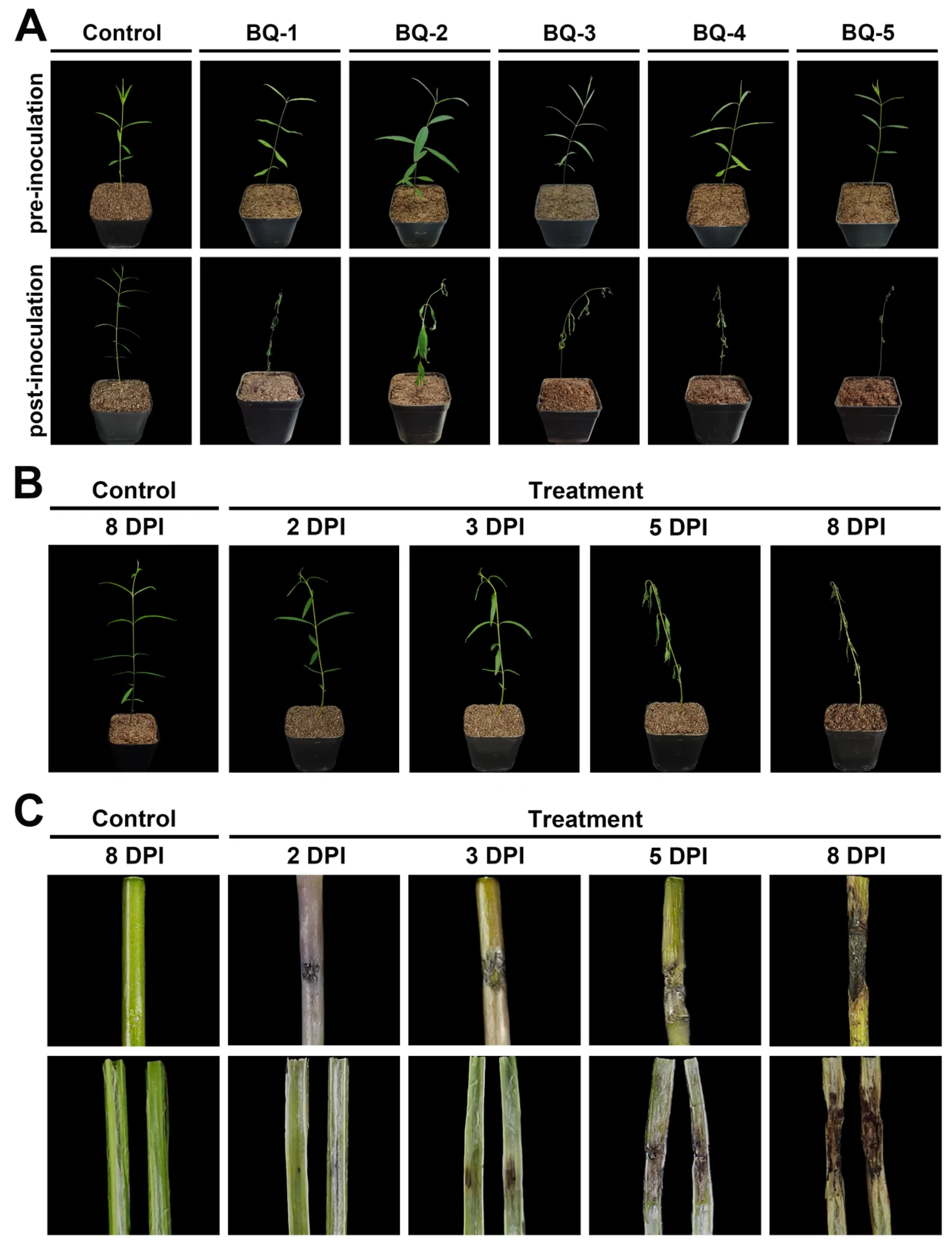
Pathogenicity assays of *V. stauntonii* wilt disease pathogens. (**A**) Pathogenicity of the five representative isolates on *V. stauntonii* in vivo. (**B**) Progressive symptom development of *V. stauntonii* wilt. Control: plants 8-day after inoculation with sterile PDA plugs. Treatment: plants at different time points after inoculation with isolate BQ-3. (**C**) Infection at the stem base of *V. stauntonii* in vivo. Control: stem base 8-day after inoculation with sterile PDA plugs. Treatment: stem base at different time points after inoculation with isolate BQ-3. DPI: days of post inoculation.

Whole-plant assays further showed that all five isolates (BQ-1 to BQ-5) induced wilting and mortality of above-ground tissues by 8 dpi (Figure 2A). Using isolate BQ-3 as a representative, the progression of wilt symptoms was observed in detail. At 2 dpi, the apical 2–4 young leaves exhibited slight wilting and drooping, with minor sunken lesions at the inoculation sites, while no obvious internal tissue damage was visible in longitudinal stem sections. At 3 dpi, wilting symptoms spread downward from the apical buds, and lesions enlarged with visible internal necrosis. By 5 dpi, most leaves exhibited wilting, curling, and shrivelling, with expanded external lesions and internal necrosis. At 8 dpi, systemic wilting had spread throughout the above-ground plant, with stem and leaf desiccation, dry rot, and tissue necrosis. External lesions encircled the stems and extended longitudinally with obvious depression; longitudinal sections revealed extensive internal damage (Figure 2B,C). In contrast, control plants remained asymptomatic. The symptom progression was consistent with that observed in the field (Figure 1C and Figure 2B). The pathogen was re-isolated from symptomatic plants, and morphological and molecular characterization confirmed its identity to the inoculated isolate.

### 3.2. Morphological Characterization and Molecular Identification of F. acuminatum Causing V. stauntonii Wilt Disease

**Figure 3:**
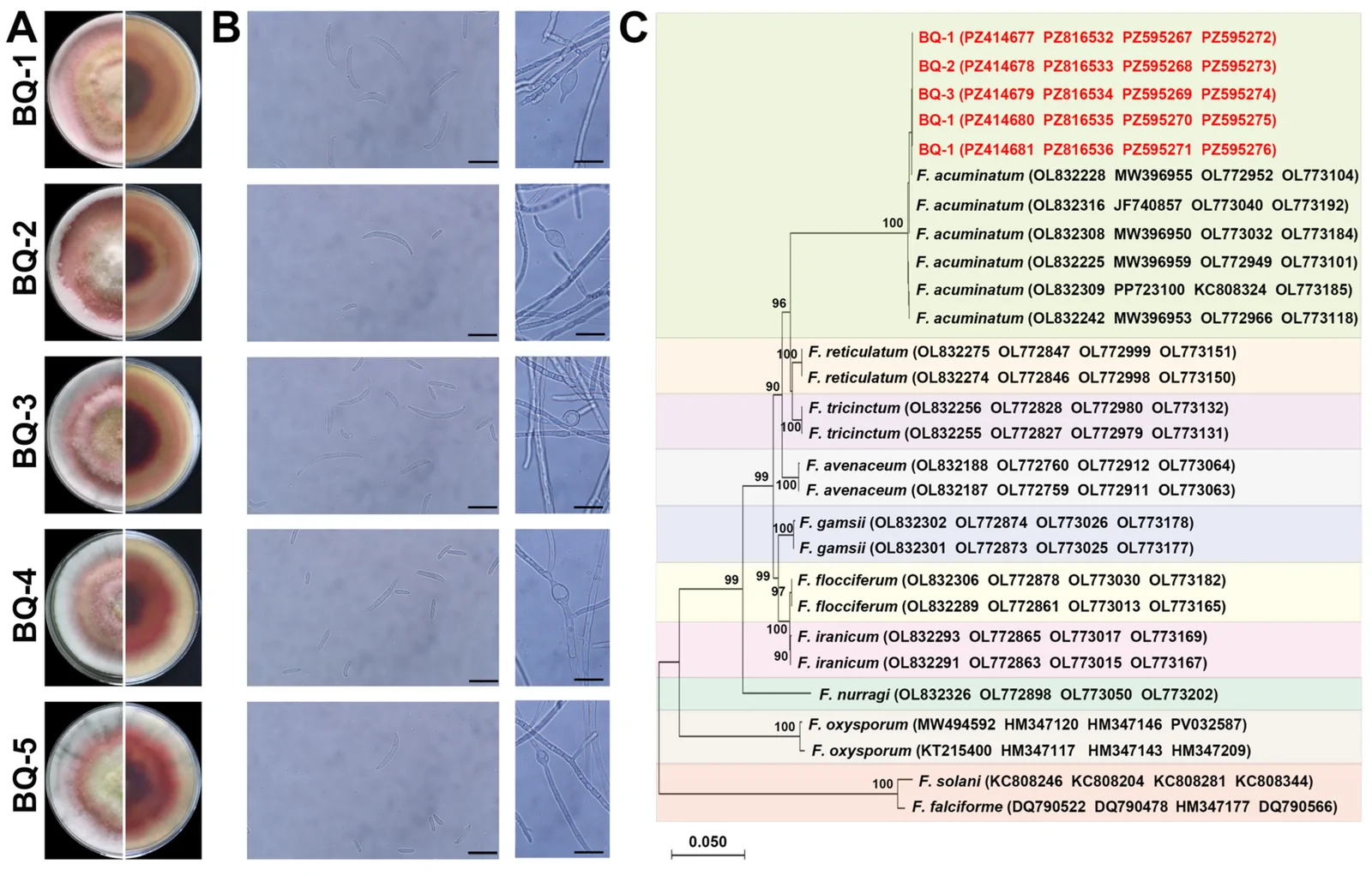
Morphological characterization and molecular identification of five representative pathogenic isolates. (**A**) Colony morphology (obverse, left; reverse, right). (**B**) Conidial characteristics (Scale bar: 20 μm). (**C**) A maximum likelihood (ML) phylogenetic tree was constructed based on concatenated sequences of the internal transcribed spacer (ITS) region, translation elongation factor 1-alpha (*TEF1-α*), RNA polymerase II largest subunit (*RPB1*), and RNA polymerase II second largest subunit (*RPB2*), with 1000 bootstrap replicates. GenBank accession numbers are listed from left to right for ITS, *TEF1-α*, *RPB1*, and *RPB2*, respectively. Strains BQ-1 to BQ-5 isolated in this study are marked in red.

All five isolates (BQ-1 to BQ-5) showed consistent growth traits and colony morphology on PDA. The isolates covered 6 cm PDA plates within 8 days, with an average growth rate of 0.65 cm/day. All colonies were circular with smooth margins and dense, fluffy aerial hyphae. Colony centers were white to pale yellow, with concentric pink-to-bright red rings in the middle layer and white margins. Pigment intensity varied slightly among isolates, but overall colony morphology was consistent (Figure 3A).

Microscopic examination revealed uniform morphological features among the five isolates. All isolates produced smooth, thin-walled conidia. Macroconidia were falcate, with acuminate apical cells and distinct basal foot cells, containing 3–5 transverse septa. Microconidia were oblong or short rod-shaped, with 0–1 septum. Chlamydospores were subglobose or ellipsoidal, mostly solitary and occasionally catenulate (Figure 3B). Conidial dimensions showed minor variations among isolates (Table 2). The macroconidia were 30.41–36.93 × 3.31–4.12 μm. The microconidia were 12.32–13.37 × 2.97–3.31 μm, and chlamydospores 8.19–8.58 × 7.79–8.27 μm. No significant morphological differences were observed among the five isolates, and all characteristics matched the description of *Fusarium* species [7].

Molecular identification was performed based on ITS, *TEF1-α*, *RPB1*, and *RPB2* gene sequences to confirm the taxonomic status of the isolates. The obtained ITS sequences of isolates BQ-1 to BQ-5 were 537, 537, 537, 538, and 536 bp, respectively. BLASTn analysis showed that these sequences shared homology rates of 99.81%, 99.81%, 100%, 99.62%, and 99.62% with the reference strains of *F. acuminatum* (GenBank accession no. MT974003, PX633852, MW016644, PP724418, PV960650), respectively. The PCR-amplified *TEF1-α* sequence (BQ-1 to BQ-5) measured 440, 441, 441, 441, and 443 bp. They were 99.31%, 99.77%, 99.77%, 99.77%, and 99.54% similar to *F. acuminatum* reference sequence (PP723100). The corresponding *RPB1* sequences (BQ-1 to BQ-5) were 1060, 966, 950, 984, and 1019 bp. These sequences exhibited sequence identities of 99.33%, 100%, 99.89%, 99.55%, and 99.57% with *F. acuminatum* strain (PV983896, LC701726, LC701727, MZ921675, LC701729), respectively. The *RPB2* sequence fragments (BQ-1 to BQ-5) were all 1000 bp. They were 99.79%, 99.89%, 99.69%, 99.90%, and 99.90% identical to the sequences of *F. acuminatum* (MW164629, OQ971920, OX346298, OX346298, PP780438), respectively. Multi-gene phylogenetic analysis showed that all five isolates clustered tightly within a single monophyletic clade with known *F. acuminatum* strains, supported by a high bootstrap value of 100% (Figure 3C). Based on consistent morphological traits and high-bootstrap phylogenetic support, these results indicate that five isolates (BQ-1 to BQ-5) are the most closely related to *Fusarium acuminatum*.

### 3.3. Biological Characteristics of F. acuminatum Causing V. stauntonii Wilt Disease

**Figure 4:**
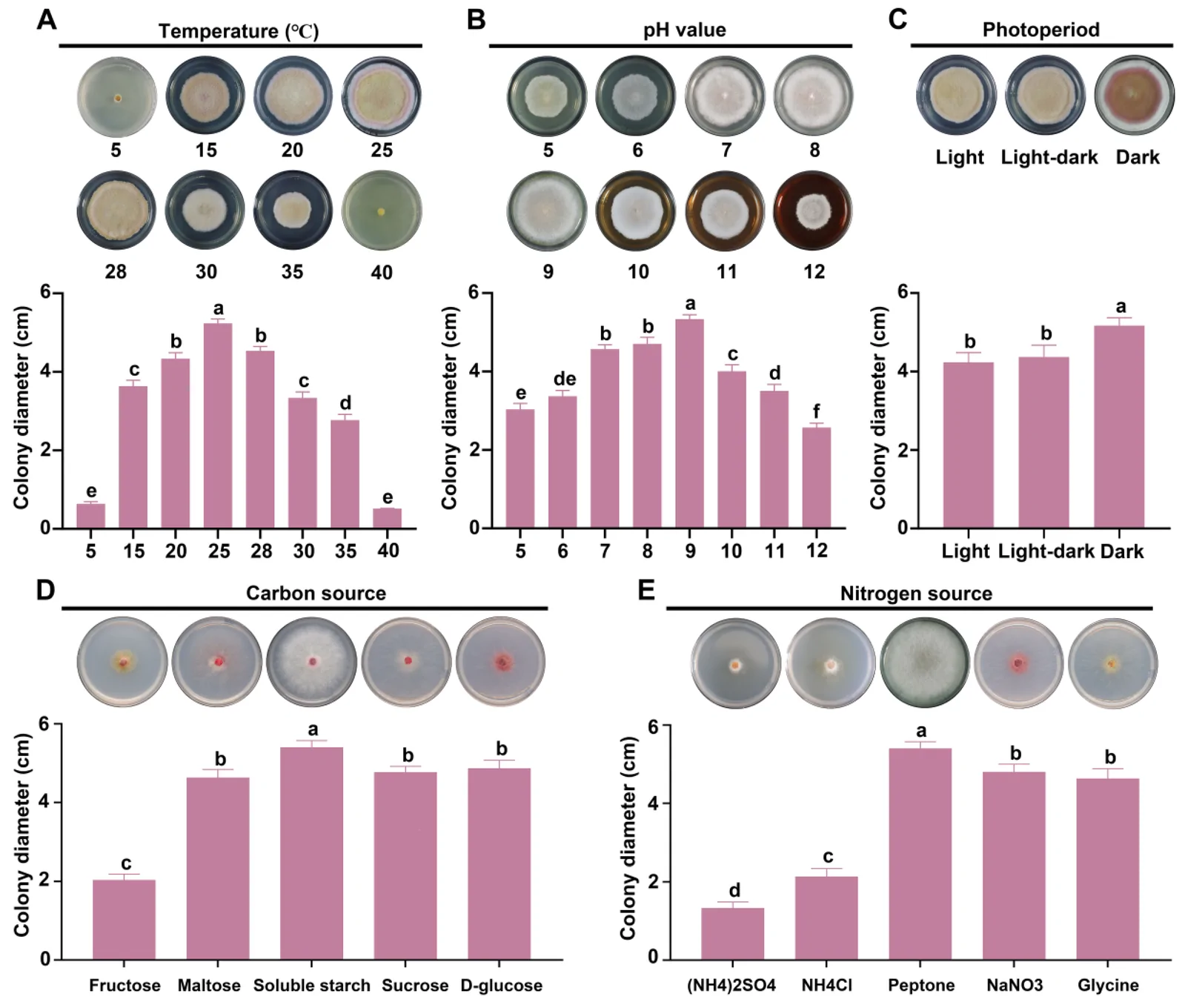
Mycelial growth of *Fusarium acuminatum* (strain BQ-3) under different conditions. (**A**) Temperatures, (**B**) pH, (**C**) Photoperiod, (**D**) Carbon source, (**E**) Nitrogen source. Different lower-case letters indicate significant differences among mean colony diameters with three biological replicates (one-way ANOVA, *p* < 0.05; Tukey’s multiple comparison test).

Temperature, pH, and photoperiod significantly affected the mycelial growth of the representative isolate BQ-3. The strain grew across a wide temperature range of 5–35 °C, with clear differences along the gradient. Mycelial growth increased rapidly from 5 °C to 25 °C and then gradually decreased as the temperature rose to 35 °C, with near-inhibition at 40 °C. Thus, 25 °C was considered the optimal temperature, with a maximum growth rate of 0.65 cm/d (Figure 4A). Similarly, pH conditions markedly affected fungal growth. The strain exhibited the slowest growth at pH 5 and pH 12, whereas neutral to weakly alkaline environments favored its development, with the fastest growth rate of 0.67 cm/d recorded at pH 9 (Figure 4B). Photoperiod had a relatively slight effect on growth; continuous light and a 12 h light/dark cycle showed no significant difference. Nevertheless, continuous darkness supported the fastest mycelial growth, with an average rate of 0.65 cm/d (Figure 4C).

Carbon and nitrogen sources markedly affected the mycelial growth and colony morphology of isolate BQ-3. Among the five carbon sources tested, soluble starch supported the fastest and densest mycelial growth (0.68 cm/d). Glucose (0.61 cm/d), sucrose (0.60 cm/d), and maltose (0.58 cm/d) also sustained stable growth but with sparser mycelia than soluble starch (Figure 4D). For nitrogen source screening, peptone was the optimal nitrogen substrate, resulting in the highest growth rate (0.68 cm/d) and the most vigorous mycelial development. In comparison, NaNO_3_ (0.60 cm/d) and glycine (0.58 cm/d) moderately supported mycelial growth, whereas (NH_4_)_2_SO_4_ showed the weakest growth-promoting effect, with a substantially lower growth rate of 0.17 cm/d (Figure 4E).

### 3.4. Preventive and Curative Effects of Ethylicin Against V. stauntonii Wilt Disease

**Figure 5:**
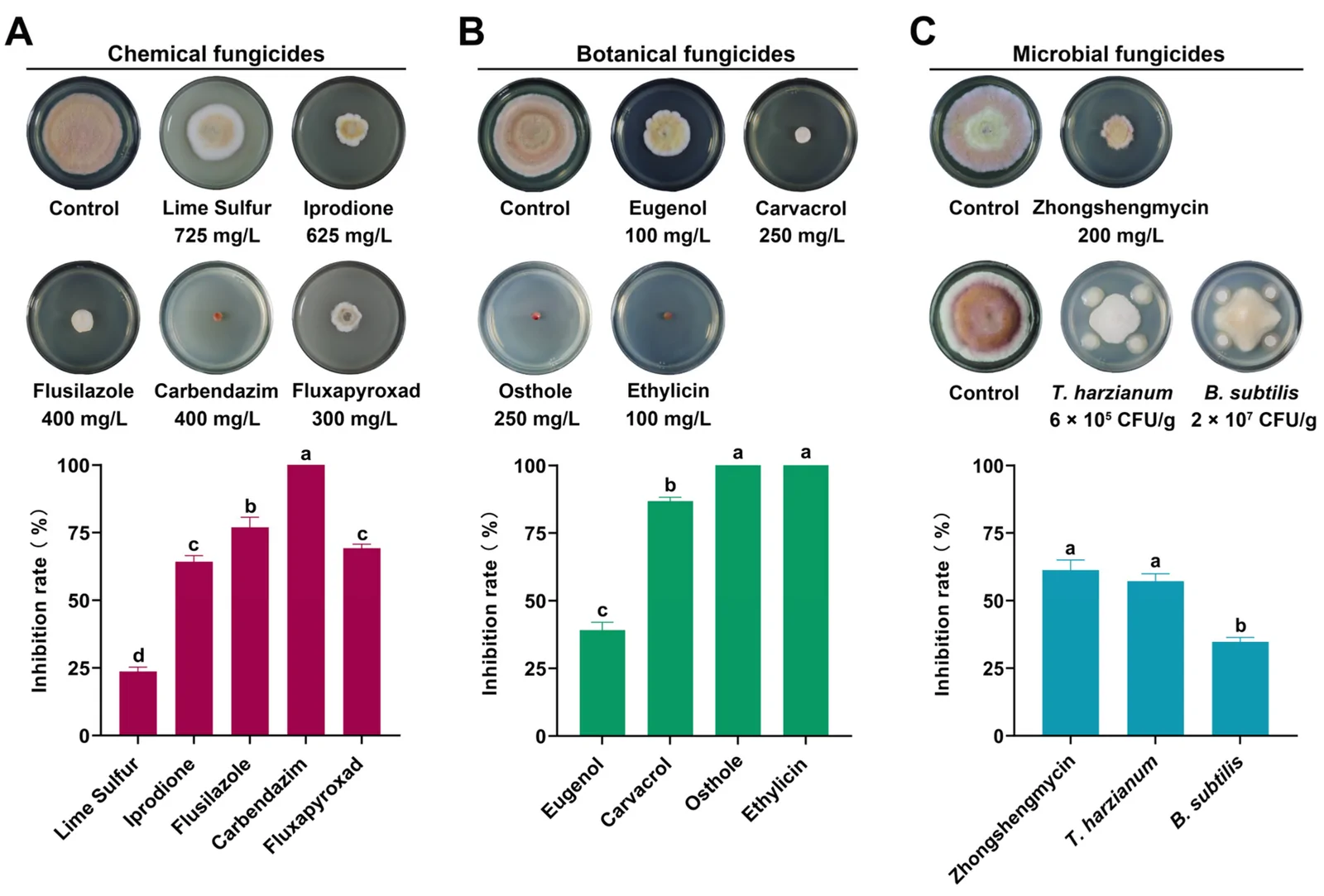
The inhibitory effects of different types of fungicides against *F. acuminatum* under various recommended dosages. (**A**) Chemical fungicides. (**B**) Botanical fungicides. (**C**) Microbial fungicides. Different lower-case letters indicate significant differences among mean mycelial growth inhibition rates with three biological replicates (one-way ANOVA, *p* < 0.05; Tukey’s multiple comparison test).

**Figure 6:**
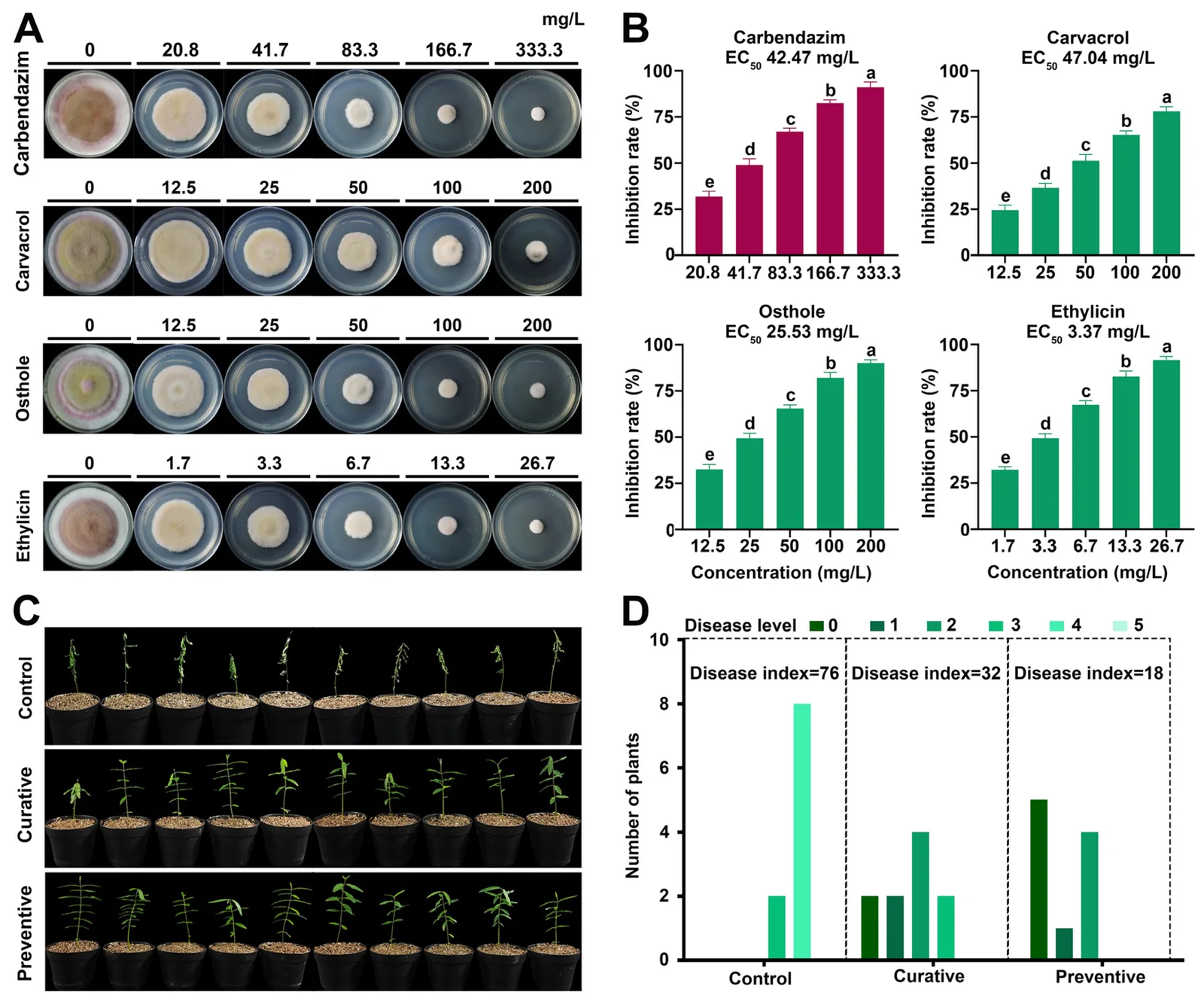
Screening of optimal fungicides for controlling *V. stauntonii* wilt caused by *F. acuminatum*. (**A**,**B**) Inhibitory effects of four candidate fungicides at different concentrations against *F. acuminatum*. Red: chemical fungicides; green: botanical fungicides. Different lower-case letters indicate significant differences among mean colony diameters with three biological replicates according to one-way ANOVA (*p* < 0.05, Tukey’s multiple comparison test). (**C**,**D**) In vivo control efficacy of ethylicin against *F. acuminatum*-induced wilt disease of *V. stauntonii*.

Twelve fungicides were screened in vitro to evaluate their inhibitory activity against *F. acuminatum*. Among chemical fungicides, carbendazim gave 100% inhibition, followed by flusilazole (76.96%), fluxapyroxad (69.21%), and iprodione (64.21%), while lime sulfur showed the lowest activity (23.63%) (Figure 5A). Among botanical fungicides, ethylicin and osthole both achieved 100% inhibition, carvacrol showed moderate efficacy (86.76%), and eugenol gave relatively low inhibition (39.11%) (Figure 5B). Among microbial fungicides, zhongshengmycin and *T. harzianum* inhibited pathogen growth by 61.27% and 57.14%, respectively, whereas *B. subtilis* exhibited the lowest inhibitory effect (34.69%) (Figure 5C). Four candidate fungicides were selected for EC_50_ determination. All agents showed a positive dose–response relationship, with inhibitory effects enhanced with increasing concentrations (Figure 6A,B). Antifungal activity against *F. acuminatum* ranked as ethylicin (EC_50_ 3.367 mg/L) > osthole (EC_50_ 25.530 mg/L) > carbendazim (EC_50_ 42.470 mg/L) > carvacrol (EC_50_ 47.043 mg/L) (Table 3). Ethylicin showed the strongest activity, indicating great potential for the green control of *F. acuminatum*-induced wilt disease.

The preventive and curative efficacies of ethylicin were further evaluated on *V. stauntonii* plants. The control group showed severe wilting, leaf shriveling, and stem desiccation. In contrast, the curative group exhibited only mild or moderate wilting, without pronounced desiccation of stems and leaves. The preventive group showed the best performance, with most plants remaining healthy and only mild apical wilting in a few plants. Disease index quantification supported the control efficacy of ethylicin (Figure 6C). The blank group had a high disease index of 76, while the preventive and curative groups achieved lower indices of 18 and 32, respectively (Figure 6D). These results indicate that ethylicin has preventive and curative efficacy against *V. stauntonii* wilt disease.

### 3.5. Ethylicin-Induced Membrane Damage and Oxidative Stress in Fusarium acuminatum Causing V. stauntonii Wilt Disease

**Figure 7:**
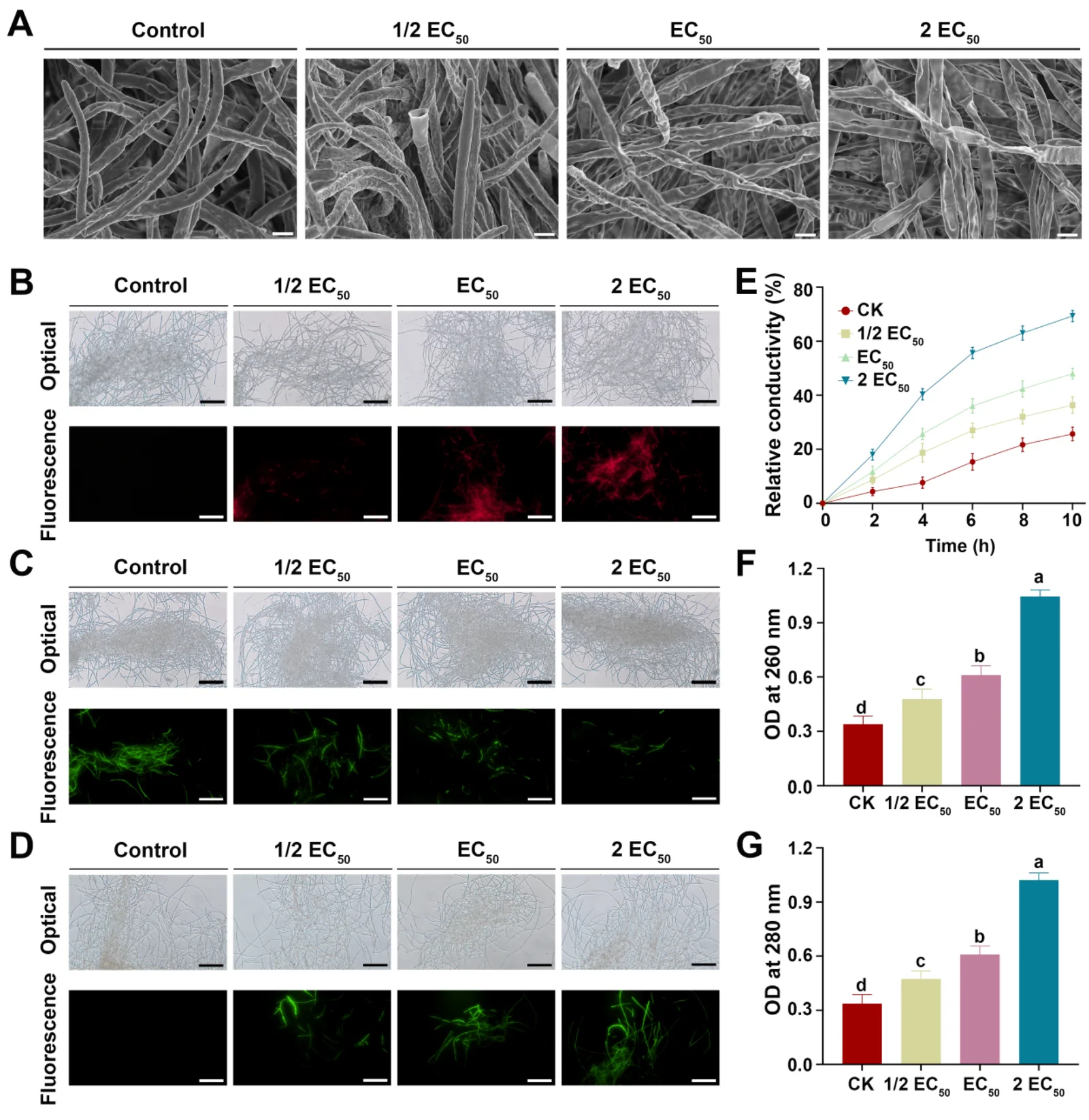
Ethylicin-induced membrane disruption and oxidative stress in *Fusarium acuminatum* mycelia. (**A**) Scanning electron microscopy of control and ethylicin-treated mycelia of *F. acuminatum* (Scale bar: 10 μm). (**B**) PI staining. PI can penetrate the membranes of dead cells and emit red fluorescence (Scale bar: 100 μm). (**C**) FDA staining. Living cells possess the ability to cleave FDA and emit green fluorescence (Scale bar: 100 μm). (**D**) ROS accumulation (Scale bar: 100 μm). (**E**) Relative electrical conductivity. (**F**) Nucleic acid leakage. (**G**) Protein leakage. Different lower-case letters indicate significant differences among the mean OD_260_ and OD_280_ values within each wavelength, respectively, with three biological replicates (one-way ANOVA, *p* < 0.05; Tukey’s multiple comparison test).

SEM was used to examine ultrastructural changes in *F. acuminatum* mycelia after 8 days of exposure to ethylicin (Figure 7A). Control mycelia showed relatively plump, regular, and intact morphology. In comparison, ethylicin-treated mycelia exhibited marked abnormalities, including evident shrinkage, surface wrinkling, and structural collapse. These effects intensified with increasing ethylicin concentrations. Membrane integrity was further evaluated by PI-FDA staining. Control mycelia showed negligible PI-derived red fluorescence, indicating intact cell membranes. In contrast, ethylicin treatment induced red fluorescence in a concentration-dependent manner, indicating membrane damage (Figure 7B). Consistently, control mycelia emitted strong and uniform FDA-derived green fluorescence, reflecting high viability and normal metabolic activity. However, ethylicin treatment attenuated and fragmented green fluorescence with increasing concentrations, suggesting reduced vitality and impaired function (Figure 7C).

Intracellular ROS accumulation induced by ethylicin was quantified using the DCFH-DA fluorescent probe (Figure 7D). Fluorescence intensity correlates positively with ROS levels. Control group exhibited only faint green fluorescence, indicating low basal ROS levels. Ethylicin treatment increased fluorescence intensity in a concentration-dependent manner, indicating excessive ROS accumulation and oxidative stress.

Membrane permeability was further assessed by measuring mycelial relative electrical conductivity, nucleic acid leakage, and protein leakage. The results showed that ethylicin significantly elevated relative electrical conductivity in a time- and concentration-dependent manner, whereas the control group remained relatively stable (Figure 7E). Similarly, nucleic acid and protein leakage were increased by ethylicin in a concentration-dependent manner. Collectively, these findings indicate that ethylicin compromises membrane integrity, induces oxidative stress, increases cell membrane permeability, and causes substantial leakage of intracellular ions, nucleic acids, and proteins.

## 4. Discussion

*V. stauntonii* is an important traditional medicinal plant widely used for the clinical management of respiratory diseases. Its rhizomes require 2–3 years of growth to accumulate sufficient dry matter and saponins before harvesting for medicinal purposes [36]. With the expansion of standardized cultivation in Xinzhou District, continuous cropping and large-scale cultivation have aggravated soil-borne disease occurrence, restricting sustainable development of the industry. This study investigated a severe wilt disease prevailing in major *V. stauntonii* cultivation regions. The causal agent was identified as *F. acuminatum* through morphology, pathogenicity assays, and multi-gene phylogenetic analysis. Previously, only *F. tricinctum* and *A. rolfsii* have been reported to cause seedling blight and southern blight on *V. stauntonii* [2,3]. This is the first report of *F. acuminatum* as the causal agent of wilt disease on *V. stauntonii*, enriching the pathogen spectrum of this host and expanding the host range of *F. acuminatum*.

Artificial in vivo inoculation recapitulated the progressive development of wilt symptoms, which closely matched the disease phenotypes observed under natural field conditions. Previous reports have described the infection process of *Fusarium* wilt, which can be outlined in three stages [37,38]. In the first stage, the pathogen may initially act as an endophyte, asymptomatically colonizing the cortical tissues [39,40]. The second stage is characterized by epidermal penetration and xylem colonization, accompanied by vessel blockage, dehydration, chlorosis, and wilting [41,42,43]. The third stage involves extensive vascular disruption, leading to progressive wilting and plant death [44,45,46]. In this study, in vivo inoculation experiments showed that at 2 dpi, no obvious internal tissue damage was observed in longitudinal stem sections. From 3 to 5 dpi, lesions gradually expanded at the inoculation site, internal necrosis became visible, and wilting symptoms spread downward from the apical bud. By 8 dpi, systemic wilting had spread throughout the above-ground plant parts, accompanied by stem and leaf desiccation, dry rot, and extensive internal tissue necrosis (Figure 2B,C). These observations appear preliminarily consistent with the previously described infection framework. It should be noted that the absence of visible internal tissue damage at 2 dpi does not necessarily indicate asymptomatic endophytic colonization. Likewise, the internal necrosis observed at 3–5 dpi does not constitute direct evidence for xylem colonization or vascular blockage. Therefore, further in-depth studies are still required to elucidate the infection process of *Fusarium acuminatum* at the vascular level.

Physiological characterization showed that the isolate grew at 5–35 °C, with optimum growth at 25 °C (Figure 4A), consistent with previous reports [15,23]. The isolate grew across pH 5.0–12.0, with optimal growth at pH 9.0 (Figure 4B). Song et al. reported vigorous growth of *F. acuminatum* at pH 11.0 [23], whereas Ding et al. found optimal growth at pH 7.0 [15]. This discrepancy may reflect host specificity or geographical differentiation of the pathogen [47]. Photoperiod experiments revealed that continuous darkness favored mycelial growth (Figure 4C). Among the tested carbon and nitrogen sources, soluble starch and peptone were the preferred sources (Figure 4D,E), consistent with earlier findings [23,48]. Most previous studies on *F. acuminatum* have focused on cash crops [49,50,51], whereas systematic research on medicinal plant isolates remains limited. These results, obtained under artificial laboratory conditions, provide baseline biological parameters for *F. acuminatum* from *V. stauntonii* and offer a preliminary reference for disease management.

Fungicide screening is critical for green prevention and precise control of emerging diseases. We screened 12 fungicides (chemical, botanical, and microbial) at their respective recommended field concentrations to preliminarily identify promising candidates against *F. acuminatum* [52,53]. Ethylicin showed the highest inhibitory activity, with the lowest EC_50_ (3.367 mg/L), outperforming carbendazim and other conventional fungicides under controlled indoor conditions (Figure 6B). Greenhouse pot assays further showed its notable preventive and curative efficacy against *V. stauntonii* wilt disease (Figure 6C). Furthermore, the observed difference in efficacy between preventive and curative treatments may be associated with the optimal application window of ethylicin. It has been previously reported that the outstanding protective activity of ethylicin is more suitable for early prevention against rice bacterial leaf blight [54]. A previous study has also reported that the protective activity of ethylicin is stronger than its curative activity against apple *Valsa* canker [55]. Similarly, the disease index of the prevention group was lower than that of the treatment group in this study (Figure 6D). Therefore, we hypothesize that preventive application could be considered as a strategy for optimizing the field application of ethylicin [54,55]. As a natural botanical fungicide, ethylicin has low toxicity, easy degradability, and low residue levels, which may mitigate the common drawbacks of long-term chemical fungicide use, including resistance development, pesticide accumulation, and reduced medicinal quality [26,56]. These properties make ethylicin a promising green candidate for sustainable disease management in medicinal crops. Therefore, further field trials are essential to assess its practical efficacy and potential application scenarios.

Preliminary mechanistic analyses revealed the basic mode of action of ethylicin against *F. acuminatum*. SEM observation suggested that ethylicin may disrupt the ordered packing of the phospholipid bilayer and weaken membrane rigidity, leading to hyphal collapse and malformation (Figure 7A) [26,56]. Since PI penetrates only membrane-damaged cells, whereas FDA is cleaved into fluorescein by viable cells [33,57], the PI–FDA dual staining showed that PI-derived red fluorescence increased and FDA-derived green fluorescence decreased with increasing ethylicin concentrations (Figure 7B,C), similar to Li et al. [32]. DCFH-DA staining revealed ethylicin-induced oxidative stress (Figure 7D), which may act synergistically with membrane damage to exacerbate cell damage [58,59,60]. Physiological tests showed that ethylicin increased relative electrical conductivity and nucleic acid and protein leakage in a concentration-dependent manner (Figure 7E–G). These findings align with accumulating evidence that membrane damage and oxidative stress may represent a common antifungal mechanism across natural and synthetic fungicides [32]. For example, previous studies have reported that ethyl ferulate, *Litsea cubeba* oil, and *Cestrum tomentosum* extracts impair membrane integrity and induce ROS accumulation in pathogens [58,59,60]. This provides a preliminary physiological basis for developing antifungal agents that target both membrane integrity and antioxidant defenses.

Despite these contributions, several limitations should be acknowledged. In both the pathogenicity and fungicide efficacy assays, we used a wound inoculation method to establish a rapid screening model for verifying pathogenicity under controlled conditions and fulfilling Koch’s postulates, while also providing a reference for preliminary assessment of the promising field candidates. However, this method does not accurately simulate the natural infection process of soil-borne vascular wilt pathogens and may affect the apparent efficacy of fungicide treatments. Therefore, these results warrant further field validation. Fungicides were compared at their recommended field rates, and the EC_50_ was used as an efficacy baseline. This approach was designed for rapid screening of the promising field candidates, which may provide timely and practical guidance for disease management, especially for emerging diseases. However, these results do not represent a direct comparison of intrinsic antifungal activities, and further dose–response trials are needed. Moreover, the EC_50_ may not reflect recommended field rates. Thus, multi-location field trials are necessary to determine the optimal concentration and timing, resistance management potential, and economic feasibility of the candidate fungicides identified in this study. In addition, the host–pathogen interaction mechanisms and primary inoculum sources of *V. stauntonii* wilt remain unclear, particularly the host signals or other factors that may mediate cortical penetration and xylem colonization. Future studies on root–pathogen interactions in *V. stauntonii* may help elucidate its root resistance and asymptomatic phenotype. This study characterized physiological alterations and oxidative stress responses induced by ethylicin in *F. acuminatum*, but the underlying molecular mechanisms remain to be further explored. These efforts will be important for developing integrated management strategies against this emerging disease.

## 5. Conclusions

This study investigated an emerging severe wilt disease affecting *V. stauntonii* in Hubei Province. Based on morphology and multi-gene phylogenetic analysis, the causal agent was identified as *Fusarium acuminatum*, representing the first record of *F. acuminatum*-induced wilt disease on *V. stauntonii*. Pathogenicity assays revealed that all five isolates were pathogenic. Biological characterization provided preliminary insights into the disease epidemiology. Fungicide screening showed that ethylicin had the strongest in vitro antifungal activity and exhibited notable preventive and curative efficacy in greenhouse pot assays. Preliminary mechanistic analysis indicated that ethylicin disrupted membrane integrity, induced excessive ROS accumulation, and caused leakage of intracellular contents. This study identified the causal agent, characterized its physiological traits, and revealed the preliminary antifungal mechanism of ethylicin, providing a theoretical basis for the sustainable management of this emerging medicinal crop disease.

## Figures and Tables

**Table 1:** Amplification sites, primer sequences, and PCR amplification parameters applied in this study.

Gene/Locus	Primer Name	Primer Sequences (5′-3′)	PCR Amplification Parameters
ITS	ITS1	TCCGTAGGTGAACCTGCGG	95 °C for 3 min; (95 °C for 30 s, 55 °C for 30 s, and 72 °C for 30 s) × 34 cycles; 72 °C for 5 min
	ITS4	TCCTCCGCTTATTGATATGC	
*TEF1-α*	EF595F	CGTGACTTCATCAAGAACATG	
	EF1160R	CCGATCTTGTAGACGTCCTG	
*RPB1*	Fa	CAYAARGARTCYATGATGGGWC	
	G2R	GTCATYTGDGTDGCDGGYTCDCC	
*RPB2*	5F2	GGGGWGAYCAGAAGAAGGC	
	7cR	CCCATRGCTTGYTTRCCCAT	

**Table 2:** Conidial sizes of the five representative pathogenic isolates.

Isolates	Macroconidia		Microconidia		Chlamydospores	
	Length	Width	Length	Width	Length	Width
	Mean (Range, SD) (µm) (n = 30)					
BQ-1	35.08 (22.17~45.27, 5.47)	4.12 (2.92~5.16, 0.60)	13.37 (5.82~18.51, 3.22)	3.31 (2.40~4.31, 0.65)	8.58 (7.43~9.58, 0.69)	8.21 (7.26~8.89, 0.52)
BQ-2	32.96 (19.76~45.14, 5.90)	3.43 (2.98~4.28, 0.34)	12.32 (5.44~16.93, 2.82)	2.97 (2.03~3.46, 0.45)	8.33 (7.69~9.33, 0.52)	8.10 (7.21~8.64, 0.50)
BQ-3	36.93 (21.65~47.18, 6.04)	3.82 (3.32~4.46, 0.33)	12.81 (5.98~17.04, 2.93)	3.20 (2.06~4.10, 0.48)	8.26 (7.58~9.13, 0.55)	8.06 (7.17~8.55, 0.44)
BQ-4	33.77 (19.32~45.90, 5.81)	3.60 (3.05~4.39, 0.34)	12.65 (6.11~16.81, 2.84)	3.11 (2.19~3.81, 0.47)	8.47 (7.54~9.36, 0.67)	8.27 (7.15~9.07, 0.58)
BQ-5	30.41 (18.99~42.96, 6.48)	3.31 (2.85~4.52, 0.42)	12.55 (6.38~16.74, 2.78)	3.03 (2.07~3.84, 0.48)	8.19 (6.94~9.42, 0.59)	7.79 (6.56~8.82, 0.38)

**Table 3:** Toxicity determinations of four candidate fungicides against mycelial growth of *Fusarium acuminatum*.

Fungicides	Toxicity Equation	R^2^	EC_50_ (mg/L)	95% CI (mg/L)
50% Carbendazim	*y* = 0.1683*x* + 42.538	0.78	42.470	33.843~51.344
5% Carvacrol	*y* = 0.2622*x* + 30.793	0.87	47.043	37.352~58.934
0.4% Osthole	*y* = 0.2737*x* + 42.663	0.79	25.530	20.134~31.079
80% Ethylicin	*y* = 2.1065*x* + 42.845	0.78	3.367	2.688~4.064

Note: *y* represents the inhibition rate; *x* represents the concentration.

## Data Availability

The original contributions presented in the study are included in the article/Supplementary Material. Further inquiries can be directed to the corresponding authors.

## Supplementary Materials

The following supporting information can be downloaded at: https://www.mdpi.com/article/10.3390/jof12090642/s1, Table S1: Names of the tested fungicides; Table S2: Concentration gradients of four candidate fungicides; Figure S1: Monthly mean air temperature and cumulative monthly precipitation in Wuhan City throughout 2025; Figure S2: Pathogenicity assays of strain BQ-1 to BQ-5 on *V. stauntonii* in vitro. DPI: days of post inoculation.
