# Supplementary material for: First Report of Fusarium acuminatum Causing Wilt Disease on Vincetoxicum stauntonii in China and the Control Strategy of Ethylicin via Membrane Damage and Oxidative Stress
Source: J Fungi (Basel). 2026 Aug 28;12(9):642. doi: 10.3390/jof12090642 (PMC13608683; doi:10.3390/jof12090642)
Supplement: Supplementary file 1 [file jof-12-00642-s001.zip › jof-4473336-supplementary.pdf]

Journal of Fungi

Supplementary Materials:

Table S1 Names of the tested fungicides.

Category	Fungicides	Formulation	Manufacturer
Chemical fungicides	30% Fluxapyroxad	SC	BASFSE (Europe)
	40% Flusilazole	EC	Shandong Topguard Crop Science Co., Ltd.
	50% Iprodione	SC	Suzhou Fumeishi Plant Protection Co., Ltd.
	50% Carbendazim	WP	Henan Yongguan Qiaodi Agricultural Technology Co., Ltd.
	29% Lime sulfur	AS	Hubei Bangmin Biotechnology Co., Ltd.
Botanical fungicides	80% Ethylicin	EC	Nanyang Xinfengda Biological Co., Ltd.
	0.3% Eugenol	SL	Henan Bisaier Agricultural Technology Co., Ltd.
	0.4% Osthole	SL	Chengdu New Chaoyang Crop Science Co., Ltd.
	5% Carvacrol	SL	Shanxi Dewei Herb Biotechnology Co., Ltd.
Microbial fungicides	3% Zhongshengmycin	AS	Sichuan Jinzhu Ecological Agriculture Co., Ltd.
	Bacillus subtilis (1×10^{10}CFU/g)	WP	Shandong Lukang Biological Pesticide Co., Ltd.
	Trichoderma harzianum (3×10^{8}CFU/g)	WP	Bayer CropScience (USA)

Note：SC = Suspension Concentrate; EC = Emulsifiable Concentrate; WP = Wettable Powder; AS = Aqueous Solution; SL = Soluble Liquid.

Table S2 Concentration gradients of four candidate fungicides.

Fungicides	Concentration gradient (mg/L)
50% Carbendazim	333.3、166.7、83.3、41.7、20.8
5% Carvacrol	200、100、50、25、12.5
0.4% Osthole	200、100、50、25、12.5
80% Ethylicin	26.7、13.3、6.7、3.3、1.7

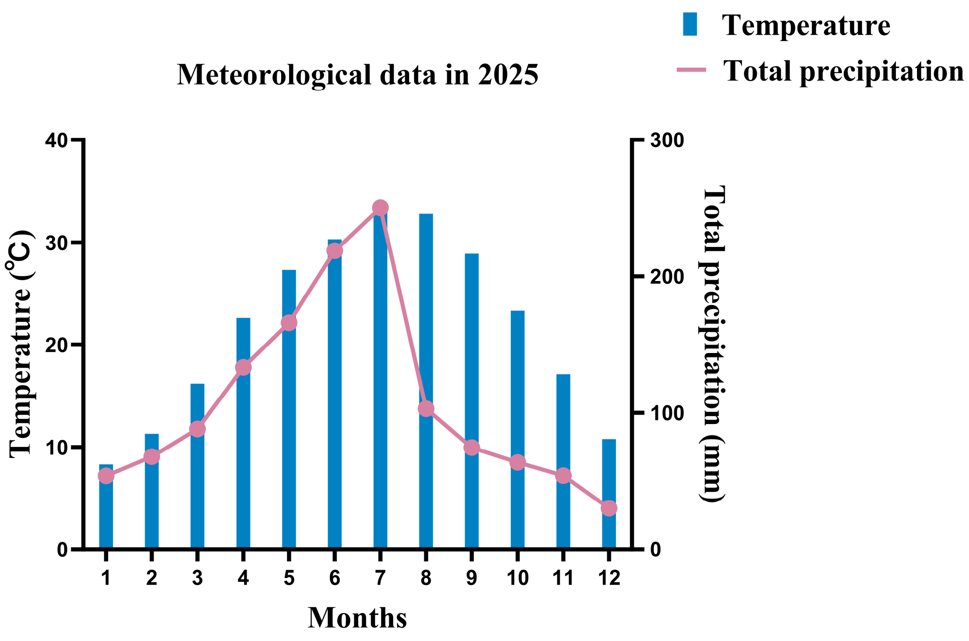

Figure S1. Monthly mean air temperature and cumulative monthly precipitation in Wuhan City throughout 2025.

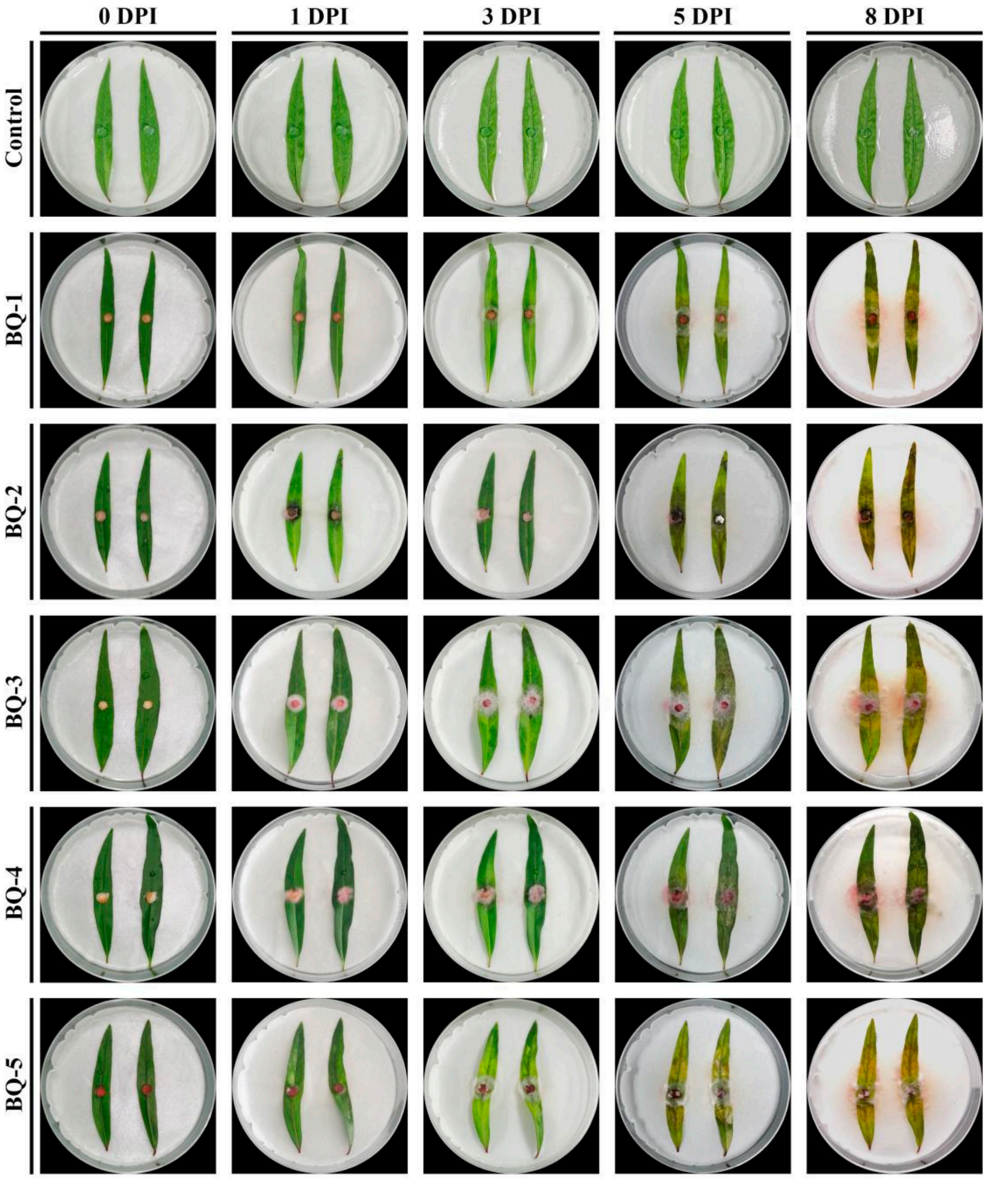

Figure S2. Pathogenicity assays of strain BQ-1 to BQ-5 on *V. stauntonii* in vitro. DPI: days of post inoculation.

https://doi.org/10.3390/jof12090642
